# Cold Blast (Attrition) Flour

**Published:** 1879-08

**Authors:** W. W. Hall


We are not the especial organ of the Health Food Company, but we rejoice in their success, and are happy to commend to our readers their efforts to supply pure and wholesome foods to the world. We daily experience the advantages which follow the use of the genuine articles prepared by this company, and can freely say that we suffer a serious disappointment whenever the table upon which our food is served lacks the appetizing and nutritious bread made from the Cold Air Attrition Flour, or the delicate, jelly-like mass which the Pearled Wheat affords, or the crisp, parched flavor of the Granulated Wheat Biscuit, or the deep ruby tint and fruity aroma of the Pomarius To us and to our family these and other choice products of the mills and ovens of the Health Food Co. have become a necessity, as we are confident they will to hundreds of thousands of other families, as soon as the merits of these superior foods are widely known. Besides, the Health Food Company were our neighbors until the demands of their growing trade compelled their removal to more spacious quarters. This proximity takes us behind the scenes and enables us to speak intelligently of their operations. We can vouch for the care which they exercise in the preparation of all their products, as well as for their promptness and trustworthiness in all business affairs. We are witnesses, also, of much of the great good which they are all the time accomplishing in the building up of broken-down bodies, and the relief of some of the most annoying and painful diseases. Nor is this all. We are in a position to testify to the great moral good accomplished by their efforts. We almost daily hear men and women bearing glad and earnest testimony to the advantages which they have experienced from the exclusive use of these pure, strong, nutritious, simple, and genuine foods, in the place of a stimulating and artificial diet. Some not only admit, but earnestly assert, that the taste for whisky and tobacco has departed under the benign influence of true foods. Our observation taught us long ago that a properly-fed person, with good digestive powers, has no uncontrollable appetite for alcoholic fluids. It is the uneasy, miserable, sour stomach which craves the biting stimulant or the poisonous sedative; which demands something, it knows not what, and accepts alcohol in some form, because that potent fluid pricks the stomach up to a new sensation while narcotizing the brain and its radiating nerves into a less vivid consciousness of stomachic misery. Seeing all the good results from the very earnest and honest work of our neighbors, why should we not say a good word, as often as we have space, for the Health Food Company? In the interest of our readers and in the advocacy of that "higher life" for humanity, for which we have so long labored, we shall continue to commend to our brother's lips the genuine foods which are here provided for all.

And now let us see what they present that is new, and upon what sort of a basis they found their claims to superior excellence. A few months ago they introduced the preparation which is known as the Cold Air Attrition Whole Wheat Flour. In our last October number we described this flour, and took occasion to object to the name given it. As the process of manufacture is described to us, there is no "attrition" whatever employed. "Attrition" means "to wear with rubbing," and in the new flouring process there is no rubbing at all. The common process of reducing the wheat-berry by grinding between stones could be described by this word more appropriately. The pulverizing power employed by our friends is that of a compressed cold-air blast, which strikes the wheat and dashes it to atoms with tremendous force. We sometime ago suggested that the term "Attrition" be dropped, and that the flour be hereafter known as the

### COLD BLAST WHOLE WHEAT FLOUR.

Of this flour we can not speak in too high terms. It is made from choice wheat, which, before pulverizing, is very carefully cleaned. In fact, its outer bark of woody fiber is removed. This is important, as the wheat-berry in its natural state is a rough, scaly structure, bristling with minute hairs and affording abundant hiding-places for the larva of insects. The pulverizing process by the cold-air blast reduces the grains to a fine powder, leaving no bran to be sifted out. Those who eat bread made from this flour get all the nutriment the wheat contains, and that, too, in a form easy of digestion. In the whole wheat meal or flour known as "Graham," even when made from sound wheat, which it is not, as a rule, the bran or hull remains in a coarse, flaky condition, and is not unfrequently a source of active and very unpleasant irritation to the stomach and bowels. Not only this, but the fact that the stomach contracts upon the contents in the natural process of digestion, and that it reluctantly and inadequately contracts when sharp and irritating points present themselves to the delicate lining membrane, shows that minute division of food is necessary to perfect digestion. The woody hull never digests, because it is not a food-substance. It is wise to get it out of our food if we can do so without extirpating some portion of the food-substance of the wheat at the same time. By the process of grinding the wheat between stones, it is impossible to retain the most nutritious part of the grain except in the form of hulls. This is because the chief nutriment lies next to the outer woody coat, and is flattened upon it by the grinding act. Sifting through fine silk bolting-cloth separates the white from the dark, the starch from the gluten and hull; that is to say, the white and heating from the dark and strength-giving food. If it were not for the hulls or woody fibers in the bran, it would be a far better food than the white part which is barreled up and sold as flour. But so long as whiteness is considered necessary, so long will our bread be nearly worthless as food. So long as mill-stones are used, so long will the bran contain the best part of the wheat. What suffering humanity needs is a fine flour containing all the food in the wheat. This is found in the Cold-Blast Whole Wheat Flour, beyond a doubt. It is equally certain that those who employ it will get more good from their bread than they have ever before secured. To such, bread will surely be, as never before, "the staff of life."

We have taken a good deal of interest in this flour and the bread and cakes made from it, and have sought information and recipes from all available sources. Some of our friends have succeeded admirably at once in baking this flour, while others have experimented several days before securing perfect results. Miss Julia Colman, who is known all over the country as a writer upon foods, gives her experience with the Cold-Blast Whole Wheat Flour and warmly commends its good qualities.

A very palatable and nutritious mush can be made from this Cold-Blast, Whole Wheat Flour, and that with scarcely any labor. We enjoy it very much when cooked at night and allowed to stand till morning, and then served cold with cream and sugar. All that is needed is to wet up the flour with as little cold water as possible, and then stir it, a little at a time, into boiling water, which must be kept over the fire and in violent ebulition, while the flour paste is being added. This destroys the taste of raw wheat as no other process will. Let it boil for fifteen or twenty minutes, and it is done. All kinds of cake, doughnuts, pie-crust—in short, anything which can be made from white flour may be made from this. We ask our lady readers to try it in all possible ways, and to write us the result, telling us at the same time, if they are willing that we should publish such accurate recipes as they may send, and print their names as authority. This will help the good cause, and serve to extend the use of what we are confident is by far the best flour in the world.

WHITE WHEAT GLUTEN.

Ten thousand mills in the United States are actively engaged in destroying our most valuable cereal—the wheat. The destructive processes are, first, grinding between hot stones, and, second, bolting or sifting out nearly all the food-portion. The Health Food Company now come to the rescue, and start one little mill in just the opposite direction. In place of the grinding and sifting and bolting, they pulverise by the cold-blast and by pounding; instead of robbing the pulverulent mass of its chief food value—its gluten and phosphorous—as do the other ten thousand millers, they eliminate its starch by careful processes, and thus provide a very delicate, gray-colored substance, full of nutriment, and which when boiled and allowed to cool assumes the form of a trembling jelly, easily digested, powerful as a builder-up of weak and delicate persons, and better capable of sustaining the life of the laborer with body or brain, than any other food we have ever seen. It is, in short, a concentrated food, being 98 per cent. gluten, and very rich in phosphatic salts. For nursing mothers and for infants it is a wonderful food. The lacteal secretion is not only increased, but is marvelously enriched by its use. It has still another value, and one of the highest importance to a large class of sufferers. *It has proved itself to possess something like a specific influence over diseases of the kidneys.* A case of diabetes has been greatly modified, and, the patient declares, absolutely cured, under our eye, within the past two months by the use of this White Wheat Gluten to the exclusion of all other food. The case was a severe one, the secretion being five gallons every twenty-four hours on the average. One of our patients, afflicted with this dangerous and devastating disease, told us he had experienced untold comfort from the use of the Gluten. It then occurred to us that the venerable sufferer from diabetes might also find relief by its use. So we ordered it at once, and gave strict injunctions to the patient and attendants that no other food should be eaten. The effect was visible in four hours in the diminished secretion, and has been followed by what seems to be a perfect cure. The theory of this gratifying result is evident, but we need not state it here We will try to discuss it in a future issue. Dr. Camplin, of London, announced it to the Royal Medical Chirurgical Society as long ago as 1862. He asserted that by freeing the wheat from starch, and retaining only the nitrogen, oil, diastase, and salts, and feeding the patient upon this rich food, the class of diseases of which we speak could be readily controlled. This we will say, here and now, that this White Wheat Gluten is absolutely the best single food we have ever tested, and is well worth a dollar a pound, instead of 25 cents. As for the manner of cooking it, perhaps the simplest and best is to make it into mush or porridge. This is done by stirring it into boiling water until thick enough, and then keeping up the boiling process for fifteen minutes. A little salt, butter, and sugar added at the close, improve the flavor. Diabetic sufferers must not eat sugar with it; all others can. Cream is always admissable, as is butter or milk. The White Wheat Gluten may be made into bread the same as "Granulated Wheat" (fine) or Cold-Blast Flour. Mixed with eggs, butter, and milk, and baked in thin cakes in a quick oven, it will be found very palatable. An excellent breakfast griddle-cake is made by taking one quart sweet milk, or milk and water, one heaping teaspoonful pure cream-of-tartar mixed dry with the Gluten, or stirred into a stiff batter of Gluten and cold water; one teaspoonful pure bicarbonate of soda dissolved in milk and stirred into the batter, and a little salt. Thin the batter with milk or water so that it will run readily from the spoon. Drop a spoonful of the batter on the griddle, having very slightly greased it first to prevent sticking. If the griddle is of soapstone, no grease will be needed. Let the cakes become brown. Sour milk works splendidly with Gluten, but the cream-of-tartar must be omitted when this is used Vinegar mixed with water, works well in the absence of cream-of-tartar.

